# Unilateral Central Retinal Artery Occlusion Revealing Giant Cell Arteritis: A Case Report

**DOI:** 10.7759/cureus.80452

**Published:** 2025-03-12

**Authors:** Hamza Lazaar, Ahmed Moumni, Anas Aziz, Taha Boutaj, Abdellah Amazouzi, Noureddine Boutimzine, Hafsa El Ouazzani, Lalla Ouafa Cherkaoui

**Affiliations:** 1 Ophthalmology, Hôpital Des Spécialités, Rabat, MAR; 2 Pathology, Hôpital Des Spécialités, Mohammed V University, Rabat, MAR

**Keywords:** central retinal artery occlusion (crao), corticosteroid, giant cell arteritis, temporal arteritis, vascular

## Abstract

Giant cell arteritis (GCA), also known as temporal arteritis, is a granulomatous vasculitis that affects large and medium-sized blood vessels. While the exact etiology remains unclear, the inflammation predominantly involves the aorta and its branches, particularly the orbital arteries. Among these, the posterior ciliary arteries (PCAs), which supply the choroid, optic nerve head, and cilioretinal artery, are especially prone to involvement. Ocular manifestations of GCA occur frequently and may result in significant visual loss. Ophthalmic conditions associated with GCA include arteritic anterior ischemic optic neuropathy (AAION), arteritic posterior ischemic optic neuropathy (NIPON), choroidal ischemia, ocular ischemia syndrome, and central retinal artery occlusion (CRAO). Diagnosis is made based on a combination of clinical, biological, and radiological findings, along with confirmation through histopathology. Early diagnosis and prompt treatment are essential to prevent permanent visual impairment or bilateral involvement. We report a case of a 62-year-old patient admitted for sudden vision loss, diagnosed with CRAO. The clinical, biological, and histopathological examination led to the diagnosis of giant cell arteritis. This study highlights the importance of early recognition and biopsy of the temporal artery for confirming the diagnosis and guiding management.

## Introduction

Giant cell arteritis (GCA), or temporal arteritis, is a granulomatous vasculitis affecting large and medium-sized vessels, primarily in individuals over the age of 50 years, with a peak incidence around 70 years of age [1]. The inflammation, the etiology of which remains undetermined, predominantly affects the aorta and its branches, and consequently, the orbital arteries [2]. Among the orbital arteries, GCA has a particular predilection for the posterior ciliary arteries (PCAs), which supply the choroid, optic nerve head, and cilioretinal artery. Ocular involvement in giant cell arteritis can affect up to 50% of cases, with visual loss occurring in nearly half of these [3].

Several ophthalmic manifestations in GCA have been described depending on the location of the involvement - arteritic anterior ischemic optic neuropathy (AAION), arteritic posterior ischemic optic neuropathy (APION), choroidal ischemia, ocular ischemic syndrome, as well as central retinal artery occlusion (CRAO) [3]. The diagnosis relies on a combination of clinical manifestations, histopathological findings from temporal artery biopsy, and/or imaging evidence of large vessel involvement [4]. It is a true ophthalmic emergency requiring rapid and appropriate treatment to prevent visual loss or bilateral involvement. We present a case of a patient admitted to the emergency department with sudden blindness of the left eye, ultimately diagnosed with central retinal artery occlusion (CRAO) secondary to giant cell arteritis (GCA).

## Case presentation

We present a case of a 62-year-old patient with no notable medical history, admitted to the emergency department for a sudden significant decrease in visual acuity in the left eye since the day before his admission. The patient also reported having experienced jaw claudication, temporal headaches, and intermittent fever two weeks prior to the onset of symptoms.

**Figure 1:**
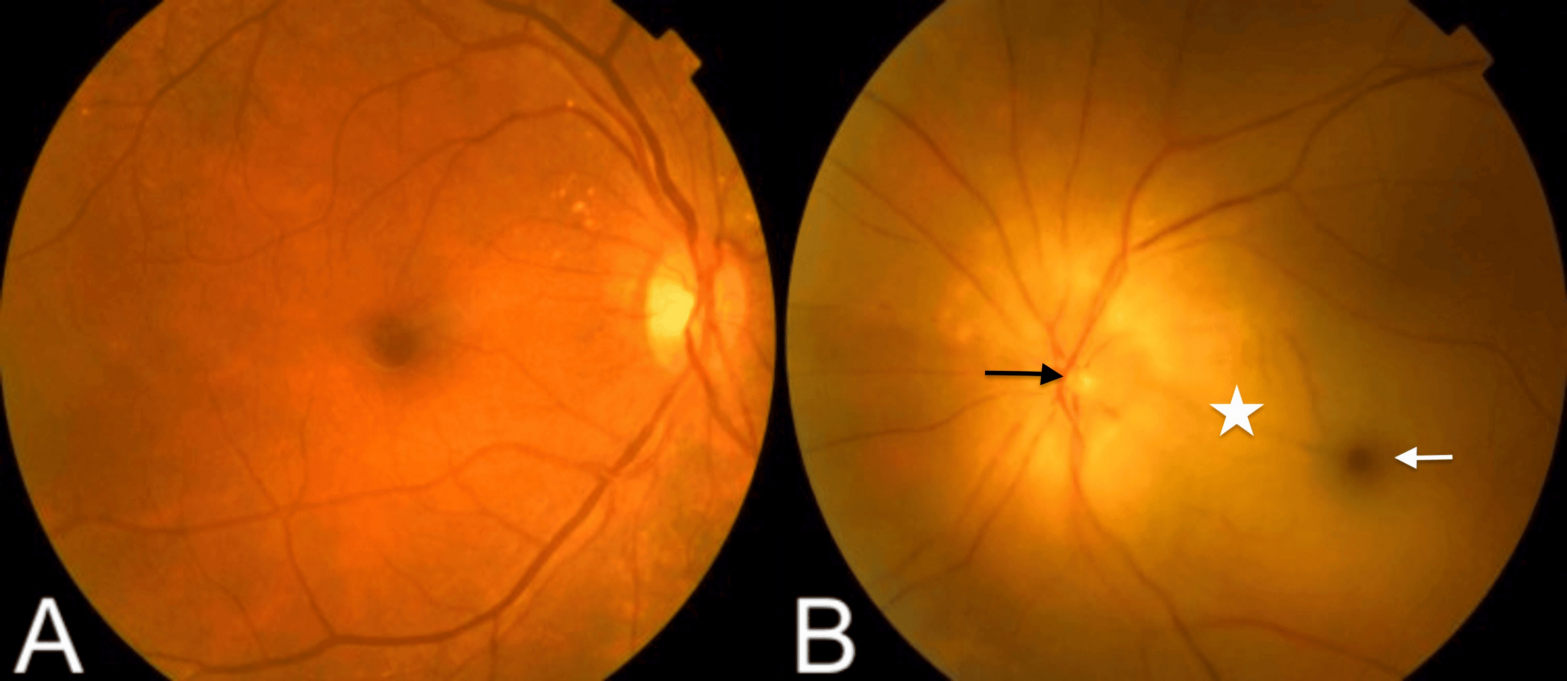
Fundus photography comparing normal and abnormal eyes. (A) Normal fundus photograph of the right eye. (B) Fundus photograph of the left eye shows an optic disc with blurred margins (black arrow), a pale retina (star), and a cherry-red spot at the fovea (white arrow).

A general examination revealed the absence of the left temporal pulse and scalp hypersensitivity. An ophthalmologic examination of the left eye found visual acuity limited to light perception, with a present relative afferent pupillary defect (RAPD). The anterior segment of the eye was unremarkable. Biomicroscopy with a slit lamp and intraocular pressure were within normal limits, fundus examination showed a pale, white retina with a thinning of the arteriolar vessels and a cherry red spot on the fovea (Figures 1A, 1B). The examination of the right eye was normal.

**Figure 2:**
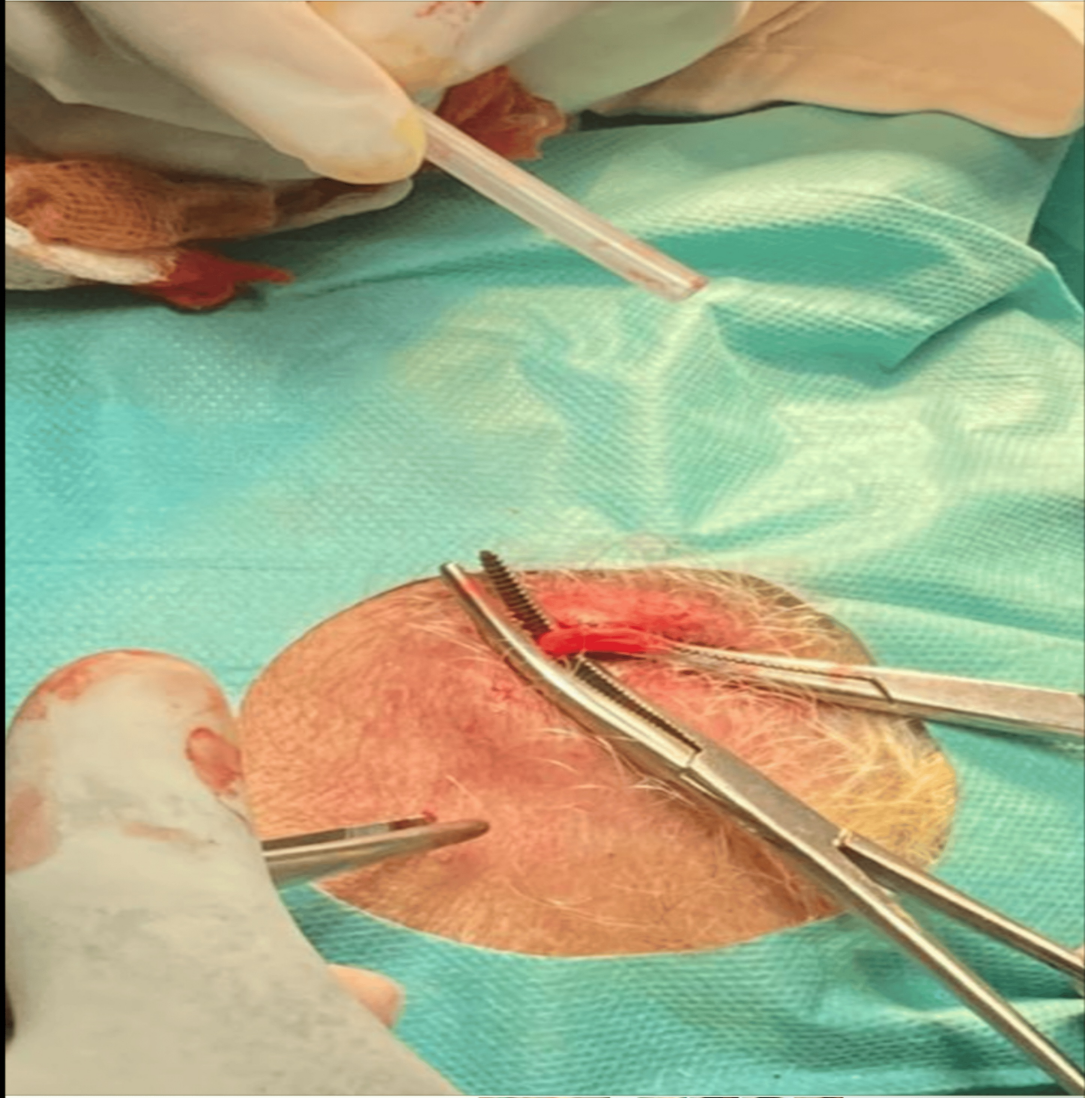
Macroscopic examination of temporal artery showing thickened and rigid superficial temporal artery with nodular areas.

Fundus fluorescein angiography demonstrated delayed arterial filling, consistent with the diagnosis of CRAO. Biological tests revealed an elevated C-reactive protein (CRP) of 130 mg/day and an ESR of 105 mm/h after 1 hour. A brain MRI to rule out a cerebrovascular accident returned normal results. A temporal artery biopsy was performed. Macroscopic examination revealed a thickened and rigid superficial temporal artery with nodular areas (Figure 2).

**Figure 3:**
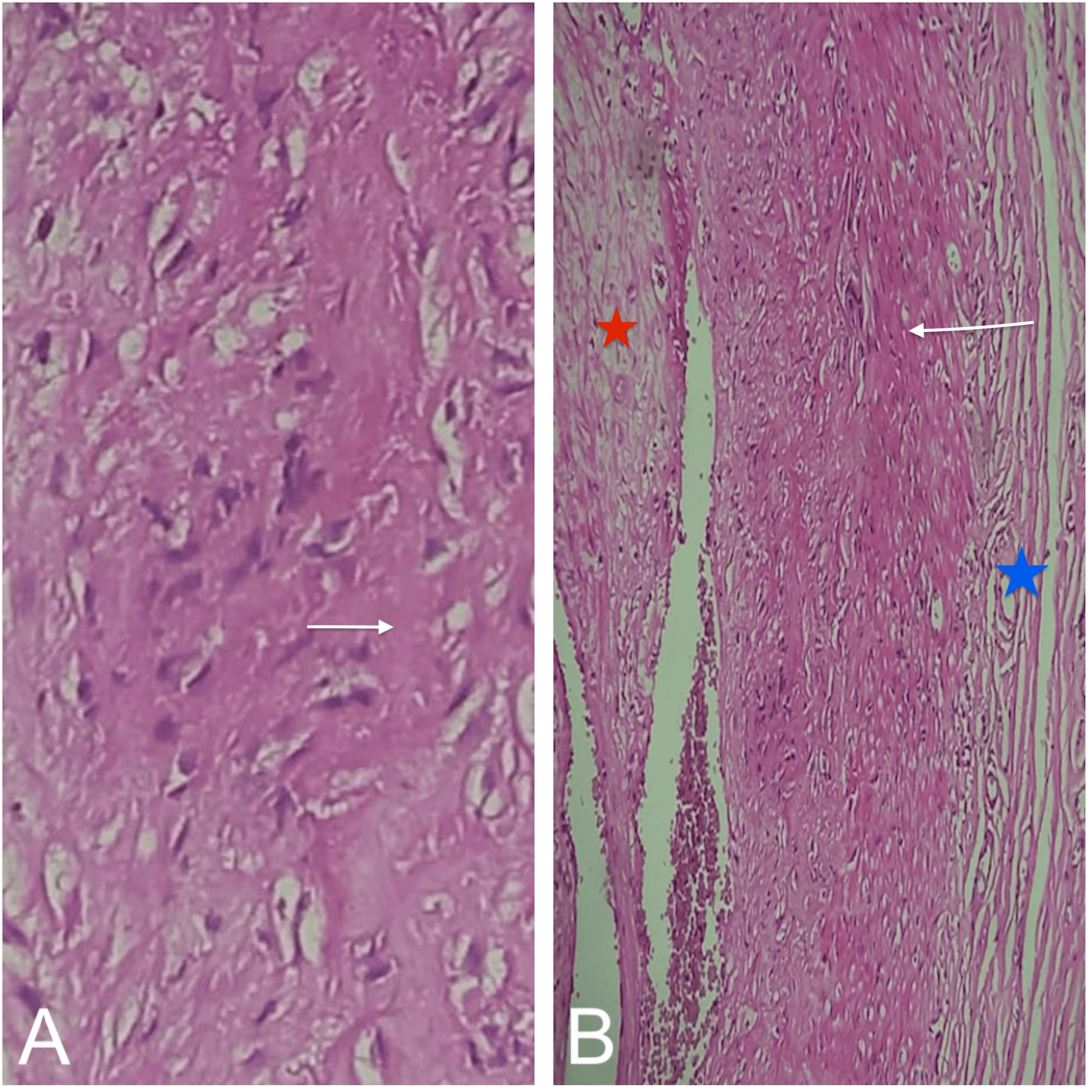
Temporal artery biopsy. (A) The media harbors epithelioid granuloma (arrow) H&E 40x. (B) microscopic image showing a vascular wall with intimal fibrous thickening (red star). Media is thickened by collagen fibrosis, with the elastic lamina (arrow) fragmentation, and the adventitia is edematous (bleu star) H&E 20x.

Histopathological examination found an arterial wall with thickened intima, the media dissociated by an inflammatory infiltrate forming two epithelioid granulomas in some areas, with the presence of a multinucleated giant cell and an edematous adventitia. The morphological features were consistent with giant cell arteritis (Figures 3A, 3B).

The patient was transferred to internal medicine, where he received an urgent IV corticosteroid bolus of 1 g/day for three days, followed by an oral corticosteroid regimen at 1 mg/kg/day until normalization of biological tests (ESR and CRP), followed by a gradual tapering over a period of one year. At the one-month follow-up, the visual acuity remained unchanged but no bilateral involvement of the central retinal artery was noted.

## Discussion

Giant cell arteritis (GCA) is a systemic vasculitis affecting the elderly, primarily targeting medium and large-caliber vessels. It is more common in women than men, with a ratio of 3:1 [5]. Non-ophthalmic clinical signs are numerous. Headaches are the most frequent manifestation, found in two-thirds of cases, often accompanied by scalp hypersensitivity [6]. Jaw claudication, which consists of mandibular pain and fatigue during chewing, is present in half of cases and is the main clinical sign associated with a positive temporal artery biopsy [7,8]. Constitutional symptoms associated with GCA are common and include fever, fatigue, weight loss, polyarthralgia, and myalgia.

Unilateral or bilateral blindness was first reported by Horton in 1934 [9]. Ocular involvement in GCA can affect up to 70% of patients, with visual loss occurring in 20% of cases [10]. Several visual impairments have been described, including central retinal artery occlusion (CRAO), as seen in the present case. In some series, CRAO is present in approximately 2-10% of cases [3]. It can be unilateral or bilateral, and some cases of CRAO sparing the cilioretinal artery have been reported [11].

CRAO is almost always associated with PCA occlusion, which is typically identified through fluorescein fundus angiography. This connection arises because the central retinal artery and one of the PCAs usually share a common trunk branching from the ophthalmic artery [3]. Other ocular manifestations include amaurosis fugax, A-AION, and A-PION.

Inflammatory biomarkers like ESR and CRP are used in the evaluation of patients suspected of having GCA. CRP is considered a more sensitive indicator than ESR in individuals undergoing temporal artery biopsy (TAB). The combined elevation of both ESR and CRP offers better specificity than either marker on its own and increases the likelihood of a positive biopsy result. Notably, in GCA patients with a confirmed biopsy, normal levels of ESR and CRP were observed in 4% of cases [12]. In the present case, the significant increase in both ESR and CRP further supported the suspicion of giant cell arteritis.

There are three primary patterns of vascular damage observed in temporal arteritis. The first, known as the classic pattern, is characterized by significant thickening of the intima and transmural inflammation. The second, referred to as the atypical pattern, may represent an intermediate stage in the resolution of the classic form. This pattern shows a less intense, non-specific arteritis with an inflammatory infiltrate composed mainly of lymphocytes, macrophages, and occasionally eosinophils and neutrophils. Moderate-to-severe intimal thickening and medial fibrosis are sometimes evident. The third pattern is the healed form, showing fibrosis in both the intima and media [13].

Treatment guidelines for giant cell arteritis (GCA) include the following recommendations

Active Disease With Visual Impairment or Cranial Ischemia

Begin treatment with high-dose intravenous corticosteroids, followed by high-dose oral glucocorticoids combined with tocilizumab, which is preferred over glucocorticoid monotherapy. Alternatively, glucocorticoids may be combined with methotrexate or used alone in some cases. After clinical remission is achieved, glucocorticoid dosage can be reduced. If remission is not attained, consider switching to an immunosuppressive agent such as abatacept or methotrexate in place of tocilizumab.

Active Disease Without Visual Impairment or Cranial Ischemia

Initiate therapy with high-dose daily oral glucocorticoids combined with tocilizumab, which is preferred over glucocorticoid-only treatment. Alternatively, glucocorticoids may be combined with methotrexate or used alone. Upon achieving remission, glucocorticoid dosage can be tapered. For those not reaching remission, immunosuppressive drugs like abatacept or methotrexate should be introduced as a replacement for tocilizumab [4].

## Conclusions

Giant cell arteritis, though rare, is a medical and ophthalmological emergency where diagnosis relies on a combination of clinical, biological, and pathological evidence. As demonstrated in the present case of central retinal artery occlusion, ocular involvement can lead to irreversible blindness without prompt treatment. Early recognition of clinical signs, such as temporal headaches, jaw claudication, and inflammatory biological changes, is crucial for initiating appropriate corticosteroid therapy. This case also emphasizes the importance of temporal artery biopsy in confirming the diagnosis and guiding treatment. A multidisciplinary approach involving ophthalmologists, internists, and pathologists is essential for optimizing clinical outcomes.

## References

[1] Mahr A, Aouba A, Richebé P, Gonzalez-Chiappe S (2017). Epidemiology and natural history of giant cell arteritis. [Article in French]. Rev Med Interne.

[2] Samson M, Corbera-Bellalta M, Audia S, Planas-Rigol E, Martin L, Cid MC, Bonnotte B (2017). Recent advances in our understanding of giant cell arteritis pathogenesis. Autoimmun Rev.

[3] Hayreh SS (2021). Giant cell arteritis: its ophthalmic manifestations. Indian J Ophthalmol.

[4] Maz M, Chung SA, Abril A (2021). 2021 American College of Rheumatology/Vasculitis Foundation Guideline for the management of giant cell arteritis and Takayasu arteritis. Arthritis Rheumatol.

[5] Gonzalez-Gay MA, Vazquez-Rodriguez TR, Lopez-Diaz MJ, Miranda-Filloy JA, Gonzalez-Juanatey C, Martin J, Llorca J (2009). Epidemiology of giant cell arteritis and polymyalgia rheumatica. Arthritis Rheum.

[6] Gonzalez-Gay MA, Barros S, Lopez-Diaz MJ, Garcia-Porrua C, Sanchez-Andrade A, Llorca J (2005). Giant cell arteritis: disease patterns of clinical presentation in a series of 240 patients. Medicine (Baltimore).

[7] Salvarani C, Muratore F (2025). Clinical manifestations of giant cell arteritis. UpToDate.

[8] Gabriel SE, O'Fallon WM, Achkar AA, Lie JT, Hunder GG (1995). The use of clinical characteristics to predict the results of temporal artery biopsy among patients with suspected giant cell arteritis. J Rheumatol.

[9] Horton BT (1934). Arteritis of the temporal vessels. Arch Intern Med (Chic).

[10] Chen JJ, Leavitt JA, Fang C, Crowson CS, Matteson EL, Warrington KJ (2016). Evaluating the incidence of arteritic ischemic optic neuropathy and other causes of vision loss from giant cell arteritis. Ophthalmology.

[11] Cohen AB, Rizzo JF (2010). Teaching neuroimages: central retinal artery occlusion with cilioretinal artery sparing in giant cell arteritis. Neurology.

[12] Kermani TA, Schmidt J, Crowson CS, Ytterberg SR, Hunder GG, Matteson EL, Warrington KJ (2012). Utility of erythrocyte sedimentation rate and C-reactive protein for the diagnosis of giant cell arteritis. Semin Arthritis Rheum.

[13] Allsop CJ, Gallagher PJ (1981). Temporal artery biopsy in giant-cell arteritis. A reappraisal. Am J Surg Pathol.

